# Supplementary material for: Multichannel optogenetics combined with laminar recordings for ultra-controlled neuronal interrogation
Source: Nat Commun. 2022 Feb 21;13:985. doi: 10.1038/s41467-022-28629-6 (PMC8861070; doi:10.1038/s41467-022-28629-6)
Supplement: Supplementary file 1 — Supplementary Information [file 41467_2022_28629_MOESM1_ESM.pdf]

Supplementary figures

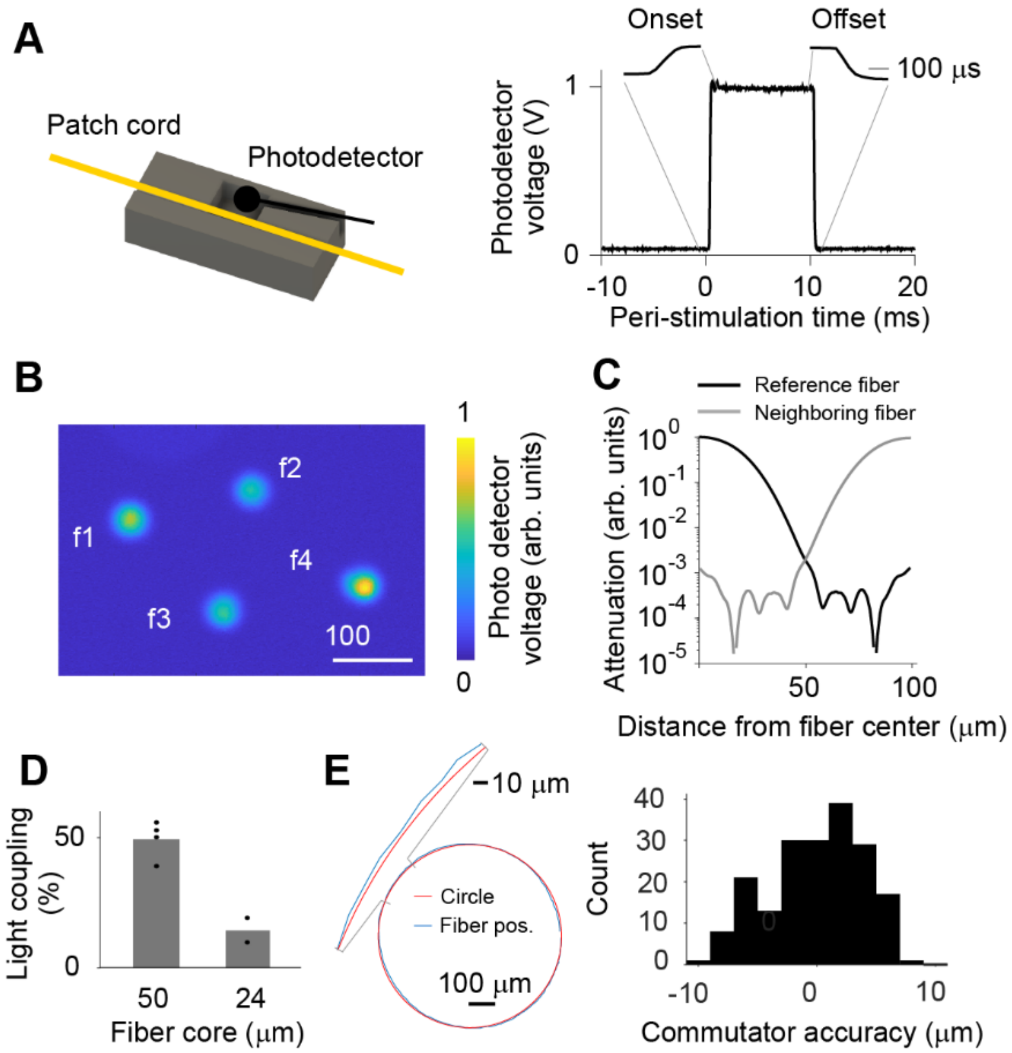

Supplementary Figure 1. Patch cord with integrated photodiode and optical commutator. A: To be able to tune the laser system in the freely moving animal and to automatically find the fiber locations, we attached a linear photodetector to the patch cord. A fraction of the light from the fibers was transmitted through the patch cord furcation tube and detected by the photodiode (left panel). The rapid targeting of the fibers was detected with the photodiode (right panel). B: Example readout from the patch cord with integrated photodiode while the galvo scanner mapped four fibers (f1, f2, f3, and f4) in the ferrule (scale bar: 100 μm). C: The optical isolation of one fiber (black line) and the mirrored version (gray line) suggests that two fibers with a separation of 100 μm can be individually addressed. D: Input laser power at ferrule divided by output power after passing the patch cord. E: Mechanical stability of the optical commutator. The red circle is the ideal rotation, and the blue circle is the center of the optical fiber. The zoomed-in section indicates minimal deviations (left). Deviation between ideal position and true fiber position (right). Source data are provided as a Source Data file.

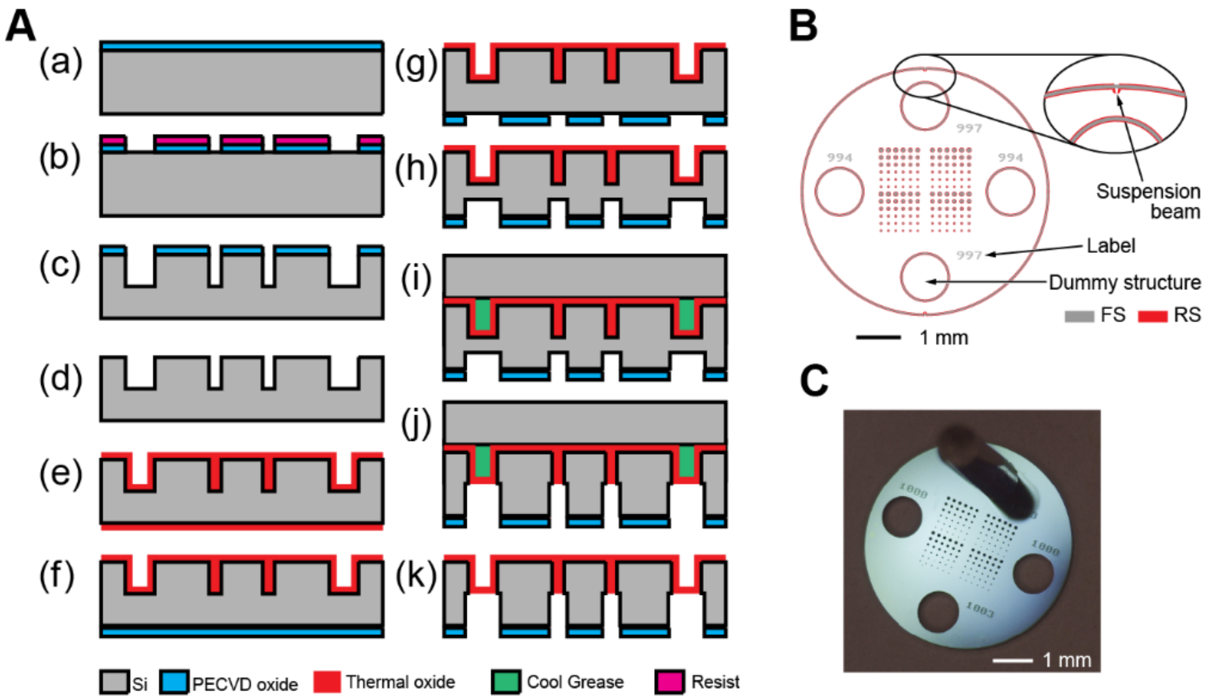

Supplementary Figure 2. Fiber matrix connector. A: Main processing steps involved in producing the Si-based connector plates of the fiber connector: (a) Deposition of PECVD SiO_x masking layer on wafer front side (FS), (b) patterning of SiO_x using lithography and RIE, (c) FS DRIE to an etch depth of 200 µm, (d) wet chemical etching of SiO_x layer, (e) thermal oxidation, (f) deposition of 2 µm of SiO_x on the wafer rear side (RS), (g) patterning of RS SiO_x, (h) DRIE to 150-µm etch depth, (i) wafer fixation on handle wafer using Cool Grease, (j) DRIE etch through, and (k) release from handle wafer. B: Layout of the lithography mask of a connector plate. The gray polygons indicate those areas patterned by DRIE from the wafer FS. The etch pattern of the wafer BS was achieved by adding the gray and red polygons. The larger outer ring defined the plate geometry. The structures on the top and bottom indicate beams that suspend the connector plate inside the silicon wafer during the entire fabrication process. Each plate with a diameter of 5 mm comprises two pairs of circular through-holes with equal diameters of approximately 1 mm. One pair of holes is used for guide pins, and the other pair is used for anchoring pins. The anchoring pins anchor the plate in the connector sleeve and the fiber bundle. C: An etched plate with guide pin. Source data are provided as a Source Data file.

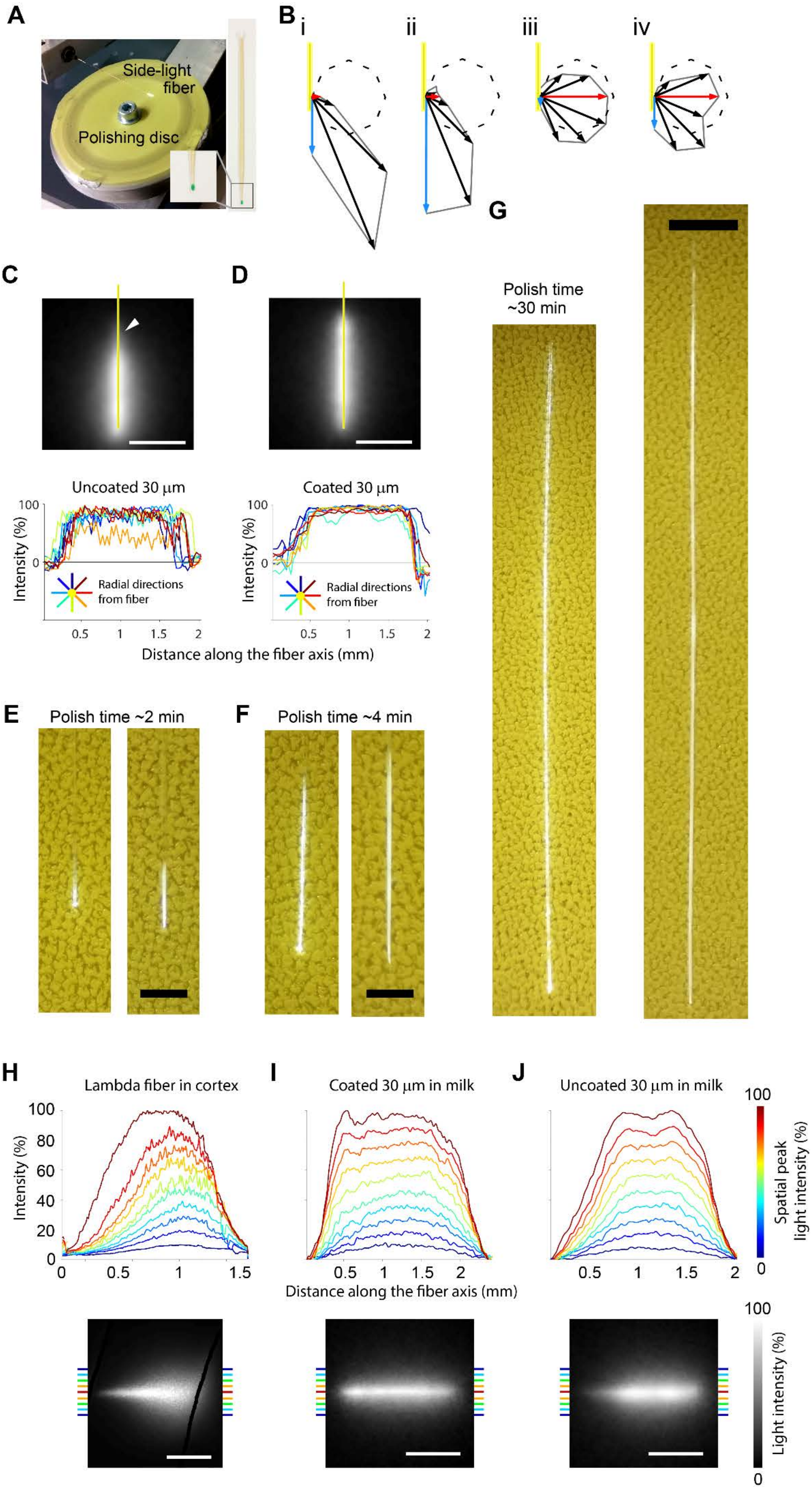
A
Side-light fiber
Polishing disc
B
i
ii
iii
iv
C
D
Uncoated 30 μm
Coated 30 μm
Intensity (%)
Radial directions from fiber
Distance along the fiber axis (mm)
E
Polish time ~2 min
F
Polish time ~4 min
G
Polish time ~30 min
H
Lambda fiber in cortex
I
Coated 30 μm in milk
J
Uncoated 30 μm in milk
Spatial peak light intensity (%)
Light intensity (%)

Supplementary Figure 3. Manufacturing and types of side-emitting optical fibers. A: Polishing plate for manufacturing side-emitting fibers. B: Emission intensities for different directions (black, red, and blue arrows) of uncoated fiber (i), uncoated fiber glued to a transparent thin film (ii), coated fiber (iii), and coated fiber glued to a transparent thin film (iv) plotted in relation to a Lambertian distribution (dashed circle). Note that the coating enhances the side-emitting component (red arrow) in relation to the forward component (blue arrow). For side-emitting fibers glued onto a transparent thin-film laminar electrode, the fiber coating ensures that the light is transmitted through the thin film (red arrow in the fourth panel) rather than to the front (blue arrow in the second panel). The dashed circle describes an ideal Lambertian emitter. C: Uncoated 2-mm fiber in milk (top). Scale bar: 1 mm. The emitted intensities at eight different angles around the probe axis as measured in air (bottom). D: Coated 2-mm fiber in milk (top). Scale bar: 1 mm. Emitted intensities at eight different angles around the probe axis as measured in air (bottom). E-G: Uncoated (left) and coated (right) fibers polished for lengths of 0.5, 2, and 10 mm, respectively. Scale bars: 500 µm, 500 µm, and 1 mm, respectively. H: Quantification of the intensity profile along a Lambda fiber in cortex at different distances from the probe. Each curve was calculated according to the distance at which the maximal intensity (along the probe axis) corresponded to a certain percentage (in increments of 10%) of the maximal intensity (at the probe). The underlying intensity map (bottom) was retrieved from Figure 2C in Pisanello et al. (2017) (scale bar: 500 µm). I: The same as in H but for a coated 30-µm fiber in milk (scale bar: 1 mm). J: The same as in H but for an uncoated 30-µm fiber in milk (scale bar: 1 mm). Source data are provided as a Source Data file.

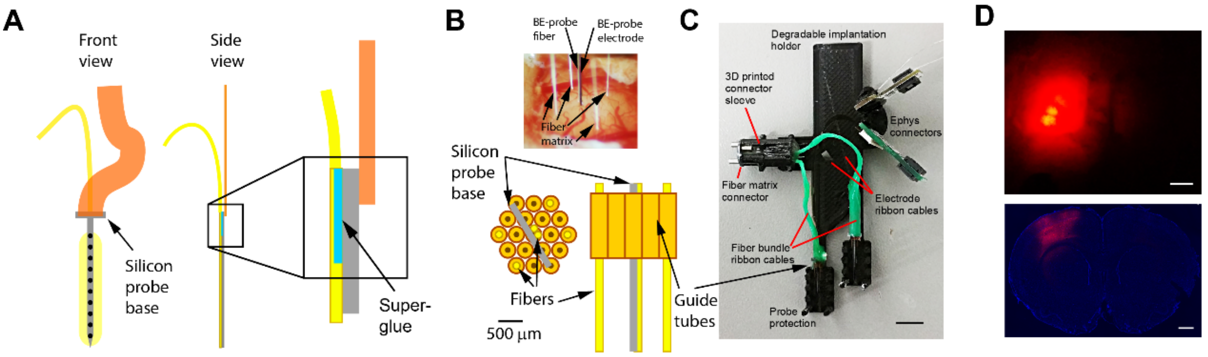

Supplementary Figure 4. Implantation of multiple fibers and electrodes. A: The fibers (yellow) were glued (blue) directly to the backside of the silicone probe (gray) at its base. This prevents interference with the ribbon cable (orange), which is typically attached to the electrode channels on the front side of the shank. B: Photo taken during surgery showing the fiber matrix (four surrounding fibers) and BE probe (white fiber next to a grayish silicon probe) (top). The fiber electrode combination allowed superficial blood vessels to be avoided, and the thinness of the fibers led to minimal dimpling upon insertion. The BE probe, consisting of a silicon electrode next to two side-emitting fibers, is surrounded by four additional side-emitting fibers (bottom). The alignment between the fibers and the probe is achieved with guide tubes (350/200-µm outer/inner diameters) in a hexagonal structure. C: Temporary implant holder for individual targeting of multiple brain areas with multiple BE probes and additional fibers. Scale bar: 5 mm. D: In vivo mCherry expression (AAV-hSyn-eNpHR3.0-mCherry-WPRE, 561-nm excitation light at 610-nm low-pass collection filter) was used to guide the implantation (upper panel, view from the top onto the brain's surface). Strong expression was detected in 6 out of 7 animals. Corresponding coronal section at Bregma 1.2 mm (lower panel). Scale bar: 1 mm. Source data are provided as a Source Data file.

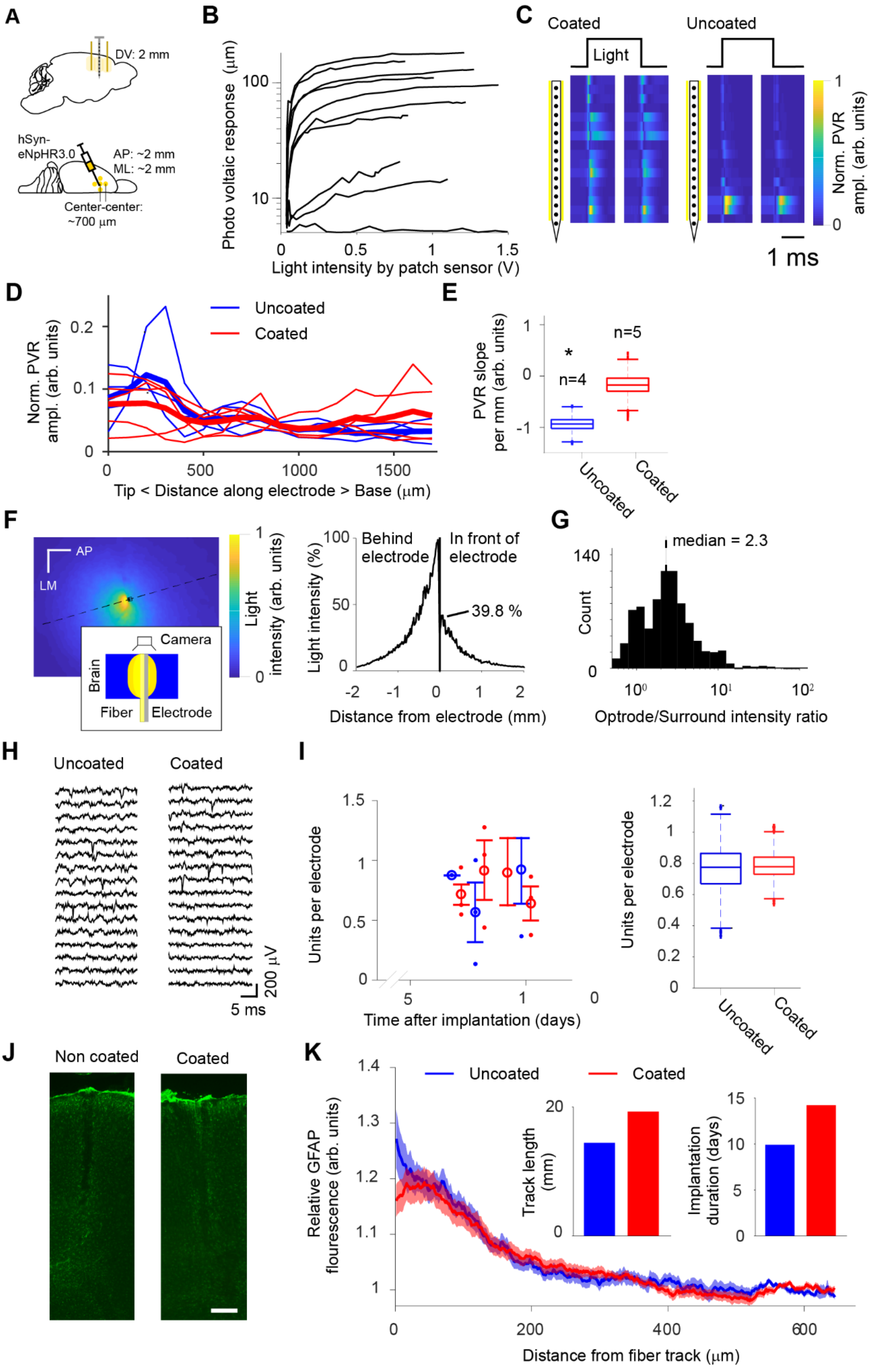

Supplementary Figure 5. BE probe performance in the freely moving animal. A: Implanted BE probes and fiber matrices. B: Assessment of BE probe functionality using PVR to ensure that the fibers are

intact. Nine of ten testable fibers gave a photovoltaic response (with the remaining four BE probes, there was a problem with the electrode ZIF-clip; see Table 1). The lower three curves represent the PVRs for the IMTEK probes, and the upper seven curves represent the PVRs for the ATLAS probes. C: Example PVR distribution along the electrode for coated and uncoated fibers. D: PVR along the electrode shank for all coated and uncoated fibers. E: Quantification of the light emission along the electrode for the uncoated and coated fibers by means of the slope of the PVR along the electrode (see panel E). A negative slope is associated with a stronger PVR toward the tip. Box-plots: central mark indicates the median, bottom and top edges refer to the 25th and 75th percentiles of the boostrapped data. F: Light distribution of a 589-nm laser source across the cortical surface from a vertical BE probe in the motor cortex of a freshly cut mouse brain (left panel). A transverse slice approximately 5 mm thick was positioned horizontally and was penetrated from below by a BE probe such that the light distribution could be imaged on the cortical surface from above (left panel, inset). Intensity cross section (dashed line in the left panel) for comparison of the light intensity at the electrodes and fibers (right panel). Scale bar: 0.5 mm. G: Comparison of PVR for BE probe fibers and fiber matrix. The relative light intensity caused by BE probe fibers is greater than that caused by the fiber matrix. H: Examples of extracellular signals for an uncoated fiber and a coated fiber. I: Quantification of electrophysiology quality as a function of days after implantation and coated (red) versus uncoated (blue) fiber–electrode combinations. n = 7 and 12 experiments with uncoated and uncoated probes, respectively. Error bars denote the standard deviation of the mean (left). Box-plots: central mark indicates the median, bottom and top edges refer to the 25th and 75th percentiles of the bootstrapped data (right). J: Representative histological examples for GFAP immunostaining (green) for uncoated and coated fibers 11 days after implantation. Scale bar: 200 µm. Those examples are representative for a total of 36 penetrations (5 animals) and 38 penetrations (5 animals) for, matrix or BE-probe, uncoated and coated fibers, respectively. K: Histological quantification of the GFAP signal with distance to the BE probe. Shaded area denotes the standard deviation of the mean. Source data are provided as a Source Data file.

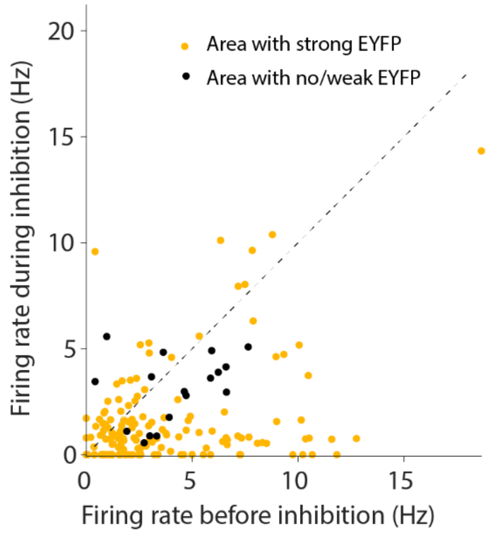

Supplementary Figure 6. Relation between baseline firing rate and firing rate during optogenetic inhibition. In areas with strong expression (yellow dots), a large proportion of neurons were inhibited (35% of the neurons were more than 90% inhibited compared to baseline activity). In areas with low expression, the inhibition effect was visibly reduced (black dots). Source data are provided as a Source Data file.

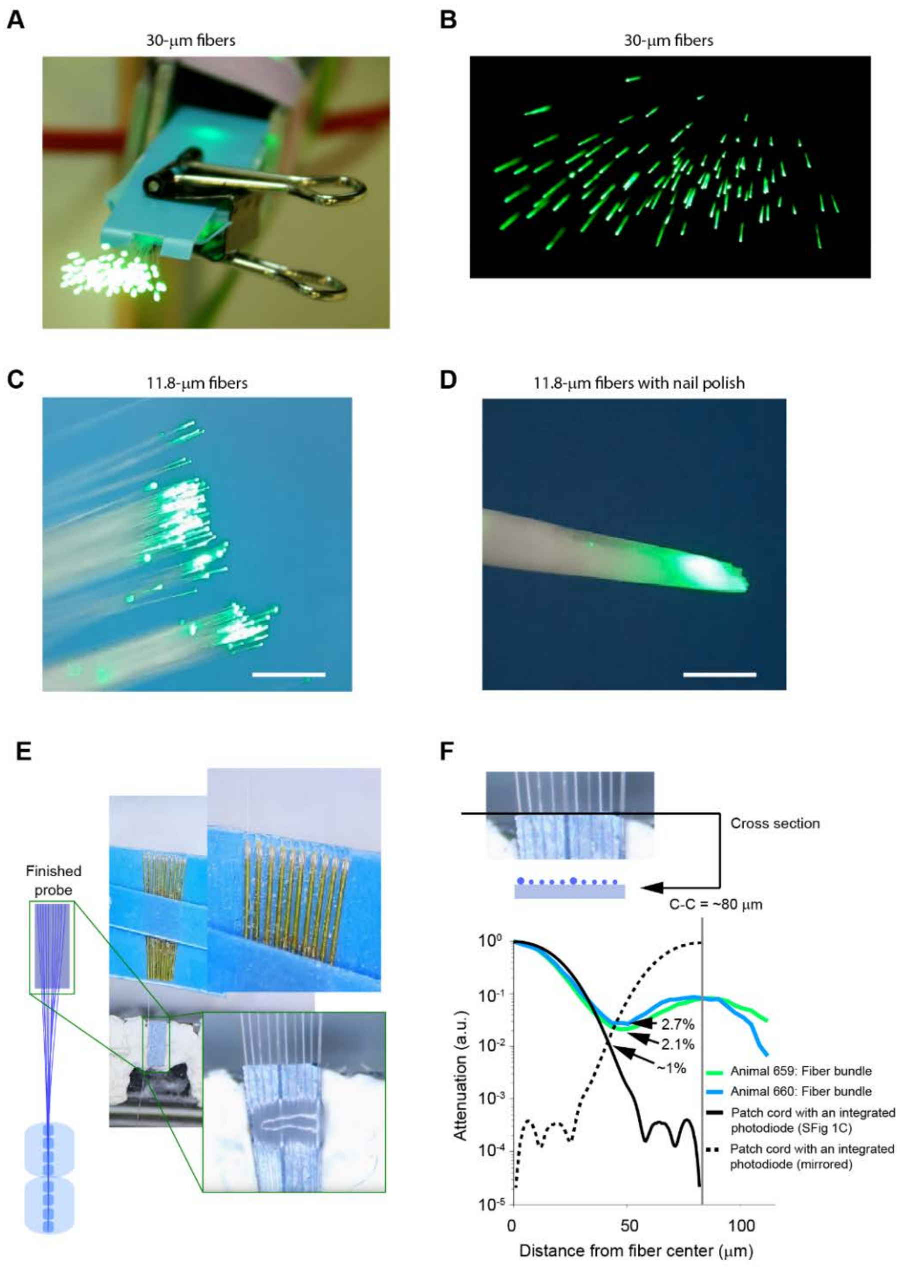

Supplementary Figure 7: Manufacturing of multifiber probes. A: Photograph of approximately 70 30-μm polished fibers held in place relative to an LED light source by a paper clip. B: Low-exposure photograph of fibers in panel A. C: Photograph of approximately 200 11.8-μm fibers. D: The fiber bundle shown in panel C is coated with nail polish to verify side emission rather than tip emission. E: Manufacturing the multifiber probe. One fiber has been inserted through the leftmost guide tube and exits at the bottom, after which it lies on top of a silicon plate (grayish plate). Ten guide tubes with beveled inlets facilitate the insertion of fibers (inset at top right). To ensure that fibers are equidistant from each other at the connector side (inset at bottom left), a tiny drop of body lotion is used to facilitate the relative adjustment of the inter-fiber spacing. F: Center-to-center fiber distance of 80 μm at connector allows minimal light leakage between fibers. If the galvo scanner positions the beam in the middle of two fibers, each fiber receives between 2.1% and 2.7% of the light as measured with the cemented photo diode. This worst-case scenario for the fiber bundle is well predicted by the attenuation retrieved from the patch cord with an integrated photodiode. Source data are provided as a Source Data file.
