# Supplementary material for: Multichannel optogenetics combined with laminar recordings for ultra-controlled neuronal interrogation
Source: Nat Commun. 2022 Feb 21;13:985. doi: 10.1038/s41467-022-28629-6 (PMC8861070; doi:10.1038/s41467-022-28629-6)
Supplement: Supplementary file 3 — Description of Additional Supplementary Files [file 41467_2022_28629_MOESM3_ESM.pdf]

Description of Additional Supplementary Files

File Name: Supplementary Movie 1
Description: Minimal brain dimpling during insertion of BE-probe and surrounding fiber matrix.
